# Management and outcomes of firearm-related vascular injuries

**DOI:** 10.1186/s13049-023-01098-6

**Published:** 2023-07-07

**Authors:** Karolina Nyberger, Eva-Corina Caragounis, Pauline Djerf, Carl-Magnus Wahlgren

**Affiliations:** 1 Department of Molecular Medicine and Surgery, Karolinska Institute, 171 76 Stockholm, Sweden; 2 Department of Trauma, Emergency Surgery and Orthopedics, Karolinska University Hospital, Stockholm, Sweden; 3 Department of Vascular Surgery, Karolinska University Hospital, Stockholm, Sweden; 4 Department of Surgery, Institute of Clinical Sciences, Sahlgrenska University Hospital, Sahlgrenska Academy, University of Gothenburg, Gothenburg, Sweden; 5 Department of Surgery, Lund University, Skåne University Hospital, Malmö, Sweden

**Keywords:** Firearm injury, Gunshot wound, Epidemiology, Vascular injury

## Abstract

**Background:**

Violence due to firearms is a major global public health issue and vascular injuries from firearms are particularly lethal. The aim of this study was to analyse population-based epidemiology of firearm-related vascular injuries.

**Methods:**

This was a retrospective nationwide epidemiological study including all patients with firearm injuries from the national Swedish Trauma Registry (SweTrau) from January 1, 2011 to December 31, 2019. There were 71,879 trauma patients registered during the study period, of which 1010 patients were identified with firearm injuries (1.4%), and 162 (16.0%) patients with at least one firearm-related vascular injury.

**Results:**

There were 162 patients admitted with 238 firearm-related vascular injuries, 96.9% men (n = 157), median age 26.0 years [IQR 22–33]. There was an increase in vascular firearm injuries over time (*P* < 0.005). The most common anatomical vascular injury location was lower extremity (41.7%) followed by abdomen (18.9%) and chest (18.9%). The dominating vascular injuries were common femoral artery (17.6%, 42/238), superficial femoral artery (7.1%, 17/238), and iliac artery (7.1%, 17/238). Systolic blood pressure (SBP) < 90 mmHg or no palpable radial pulse in the emergency department was seen in 37.7% (58/154) of patients. The most common vascular injuries in this cohort with hemodynamic instability were thoracic aorta 16.5% (16/97), femoral artery 10.3% (10/97), inferior vena cava 7.2% (7/97), lung vessels 6.2% (6/97) and iliac vessels 5.2% (5/97). There were 156 registered vascular surgery procedures including vascular suturing (22%, 34/156) and bypass/interposition graft (21%, 32/156). Endovascular stent was placed in five patients (3.2%). The 30-day and 90-day mortality was 29.9% (50/162) and 33.3% (54/162), respectively. Most deaths (79.6%; 43/54) were within 24-h of injury. In the multivariate regression analysis, vascular injury to chest (*P* < 0.001) or abdomen (*P* = 0.002) and injury specifically to thoracic aorta (*P* < 0.001) or femoral artery (*P* = 0.022) were associated with 24-h mortality.

**Conclusions:**

Firearm-related vascular injuries caused significant morbidity and mortality. The lower extremity was the most common injury location but vascular injuries to chest and abdomen were most lethal. Improved early hemorrhage control strategies seem critical for better outcome.

## Introduction

Gun violence is a global increasing health problem not only affecting the livelihood of those injured but also places a burden on healthcare systems and societies [1]. Firearm injuries, particularly to the vasculature, have been associated with high morbidity and mortality [2–4]. Data from the National Inpatient Sample in the United States showed that firearms injuries with vascular repair were independently associated with higher injury severity score and mortality [5]. The most common injury location of firearm injuries are the lower extremities, and those injuries requiring vascular repair have been associated with higher rates of amputation [6, 7]. However, there are few contemporary nationwide studies and there is still a lack of evidence on prehospital and in-hospital management of firearm-related vascular injuries. Our aim was therefore to analyse population-based epidemiology of vascular injuries from firearms including demographics, management, and patient outcomes.

## Methods

### Study population

This was a retrospective nationwide epidemiological study including all patients with firearm-related vascular injuries from the national Swedish Trauma Registry (SweTrau) from January 1, 2011, to December 31, 2019 [8]. There were 71,879 trauma patients registered during the study period of which 1010 patients were identified with firearm injuries (1.4%). There were 162 patients (162/1010; 16%) with at least one firearm-related vascular injury which constituted the final study cohort. The study was approved by the Ethical Review Agency (2019-05863).

### Study aims

The primary aim was to investigate the national epidemiology of firearm-related vascular injuries during the study period. Secondary aims were to assess anatomical distribution of injuries, operative procedures, and patient outcome.

### Data sources

Data were extracted from the national trauma registry, SweTrau [7]. SweTrau, started in 2011, is the only nationwide trauma database, covering 84% of the trauma receiving hospitals in Sweden. The SweTrau database follows “*The Utstein Trauma Template for Uniform Reporting of Data Following Major Trauma; Data Dictionary*”, which represent a uniform set of variables considered most important for comparing trauma systems and outcomes in Europe [9]. Data access for this study was approved by the registry steering committee.

### Inclusion and exclusion criteria

Patients of all ages and genders, admitted with firearm injury and at least one diagnosed vascular injury, were included in the study (n = 162). A firearm injury was defined as shot by handgun, shotgun, rifle, or other firearm of any caliber. A vascular injury was defined with a registry code for vascular injury from the International Classification of Diseases, tenth revision (ICD-10). Patients admitted to the reporting hospital more than 24 h after injury and patients declared dead before hospital arrival were excluded as recommeded by the Utstein consensus process [9]. At least one of the following inclusion criteria was needed for registration in Swetrau: trauma team activation, New Injury Severity Score (NISS) > 15 or NISS > 15, and transferred from another hospital within 7 days. Injuries that were not caused by firearms and did not include a registered vascular injury were excluded from further analysis (n = 71 717). Missing values for specific variables were imputed for some variables and are reported below.

### Statistical analysis

Data were presented as median with interquartile range (IQR). Descriptive statistics were performed for patient characteristics and outcomes. Univariate analysis and multivariable logistic regression analyses were performed to identify risk factors for 24-h and 30-day mortality post trauma admission. In the logistic regression model age, gender, vascular injury location (femoral artery, carotid artery, thoracic aorta) and anatomical injury location (head, face, neck, chest, abdomen, abdomen, spine, upper extremity, lower extremity and unspecified) were included as covariates. The Poisson regression model was used to analyse trauma trends over the years. *P*-value < 0.05 was considered significant. Analysis was performed with IBM® SPSS Statistics V27.0.

## Results

### Patient demographics

**Fig. 1:**
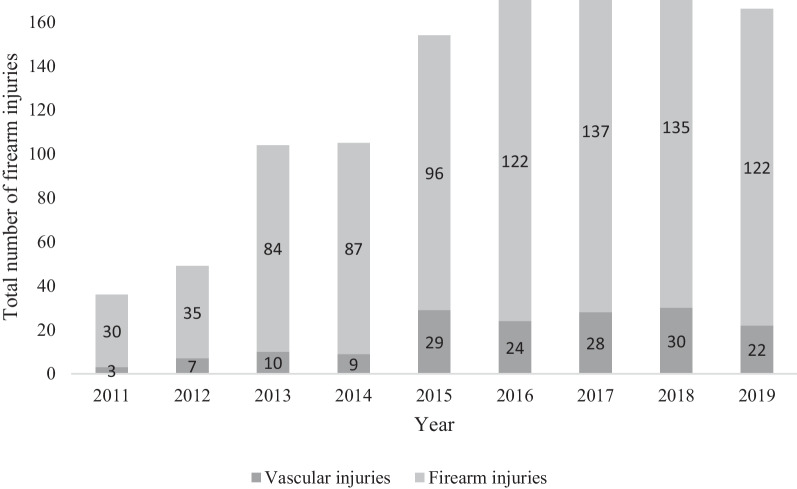
Distribution of vascular firearm injuries and firearm injuries overall between 2011 and 2019. There was an increase in vascular firearm injuries over time (*P* = 0.005)

There were 162 patients admitted with 238 firearm-related vascular injuries, 96.9% men (n = 157) and 3.1% women (n = 5), median age 26.0 years [IQR 22–33]. There was an increase in vascular firearm injuries over time (*P* < 0.005) (Fig. 1). The median ISS and NISS were 17.5 [IQR 10–32.75] and 27 [IQR 16–48], respectively. Firearm related vascular injuries dominated in the younger population where the age group 21–30 years (n = 96) was most common (*P* = 0.011), followed by age group 31–40 years (n = 28) (Table 1). There were few vascular injuries in the population > 60 years (n = 3).

**Table 1:** Vascular firearm injuries in different age groups over time (n = 162 patients)

Year	Age group							
	< 20 years	20–30 years	31–40 years	41–50 years	51–60 years	61–70 years	> 70 years	Total
2011	0	2	1	0	0	0	0	3
2012	1	2	2	2	0	0	0	7
2013	0	6	2	2	0	0	0	10
2014	1	3	1	2	2	0	0	9
2015	3	17	4	1	3	1	0	29
2016	2	16	1	2	3	0	0	24
2017	1	21	5	0	0	0	1	28
2018	2	16	9	1	2	0	0	30
2019	2	13	3	3	0	1	0	22
Total	12	96	28	13	10	2	1	162
*P*-value	0.064	0.011	0.081	0.56	0.544	0.268	0.476	0.005

### Clinical data

Prehospital and emergency department (ED) vital signs are displayed in Table 2. Prehospital and ED systolic blood pressure (SBP) < 90 mmHg or no palpable radial pulse was seen in 48.7% (58/119) and 37.7% (58/154) of patients, respectively. There were 28.5% (37/130) prehospital cardiac arrests. Prehospital and ED intubation was performed in 23.4% (30/128) and 41.8% (64/153), respectively. The most common prehospital transportation was ground ambulance 69.1% (112/162); 9.3% (n = 15) arrived with helicopter, 6.2% (n = 10) with private transportation, 1.8% (n = 3) with police, 0.6% (n = 1) with other transportations, and 13.0% (n = 21) had unknown transportation mode. The median time from the trauma alarm to arrival at the hospital was 35 min [IQR 26–50] (n = 153).

**Table 2:** Prehospital and emergency department clinical data (n = 162 patients)

Clinical data		
ISS*	17.5 [10–32.75]	162
NISS*	27 [16–48]	162
NISS ≤ 15	9.5 [6–12]	38
NISS 16–24	19 [17–22]	33
NISS ≥ 25	41 [34–58]	91
Prehospital SBP (mmHg)*	108 [71–130]	74
Prehospital GCS*	15 [3–15]	103
Prehospital RR/minute*	20[18–24.5]	59
ED SBP (mmHg)*	120 [90.5–135.75]	122
ED GCS*	15 [13–15]	123
ED RR/minute*	20 [17–21]	77

*Median [IQR]; *ISS* Injury Severity Score, *NISS* New Injury Severity Score, *ED* emergency department, *SBP* Systolic blood pressure, *GCS* Glasgow Coma Scale, *RR* respiratory rate

### Vascular anatomical injury data

The anatomic locations of vascular injuries are presented in Table 3. The most common anatomical vascular injury location was lower extremity (41.7%) followed by abdomen (18.9%) and chest (18.9%). The dominating vascular injury was the common femoral artery (17.6%, 42/238) followed by the superficial femoral artery (SFA) (7.1%, 17/238) in the lower extremity. In the abdomen, the iliac arteries (7.1%, 17/238) and the inferior vena cava (5.5%, 13/238) were the most frequent vascular injuries. In the chest, injuries to the thoracic aorta (6.7%, 16/238) were most common.

**Table 3:** Anatomical locations of vascular injuries (n = 238)

Anatomic vascular injury location	N (%)
*Lower extremity vessels*	
Common femoral artery	42 (18)
Deep femoral artery	1 (0.4)
Superficial femoral artery	17 (7.1)
Popliteal artery	12 (5)
Tibioperoneal trunc	1 (0.4)
Anterior/posterior tibial artery	3 (1.3)
Peroneal artery	1 (0.4)
Femoral vein	15 (6.3)
Popliteal vein	2 (0.8)
Miscellaneous lower extremity vessel	13 (5.5)
*Upper extremity vessels*	
Innominate/subclavian artery	7 (2.9)
Innominate/subclavian vein	3 (1.3)
Axillary artery	2 (0.8)
Brachial artery	5 (2.1)
Radial/ulnar artery	4 (1.7)
Axillary/brachial vein	2 (0.8)
Miscellaneous upper extremity vessel	6 (2.5)
*Abdominal vessels*	
Abdominal aorta	5 (2.1)
Inferior vena cava	13 (5.5)
Mesenteric artery/celiac trunk	3 (1.3)
Iliac artery	17 (7.1)
Iliac vein	2 (0.8)
Splenic/gastric artery	2 (0.8)
Portal vein	1 (0.4)
Mesenteric vein	2 (0.8)
Miscellaneous abdominal vessel	5 (2.1)
*Thoracic vessels*	
Thoracic aorta	16 (6.7)
Pulmonary vessel	7 (2.9)
Superior vena cava	3 (1.3)
Intercostal artery	3 (1.3)
Miscellaneous thoracic vessel	1 (0.4)
*Head and neck vessels*	
Common carotid artery	3 (1.3)
Internal carotid artery	3 (1.3)
Carotid artery unspecified	3 (1.3)
Vertebral artery	3 (1.3)
Jugular internal/external vein	6 (2.5)
Facial/temporal artery	4 (1.7)

Vascular injuries were distributed between 66.0% arterial injuries (157/238) and 18.1% venous injuries (43/238); 15.9% were not specified arterial or venous injury (38/238); 19.1% of patients (31/162) had combined arterial and venous injuries. About one third of patients (36.4%, 59/162) had multiple vascular injuries, and 8.6% (14/162) had multiple vascular injuries in more than one anatomic injury location.

In the 58 patients (97 registered vascular injuries) with prehospital SBP < 90 mmHg or no palpable radial pulse, the distribution of anatomical vascular injury location was thorax 36.0% (35/97), abdomen 25.8% (25/97), lower extremity 25.8% (25/97), and neck 12.4% (12/97). The median transportation time for these patients (n = 50) was 31 min [IQR 23.3–47.8]. The most common vascular injuries in these patients were thoracic aorta 16.5% (16/97), femoral artery 10.3% (10/97), inferior vena cava 7.2% (7/97), lung vessels 6.2% (6/97) and iliac vessels 5.2% (5/97).

### Vascular surgery procedures

There were 74 patients (7.3% of patients with firearm injuries; 74/1010) that required one or more vascular surgery procedures (n = 156 registered vascular surgery procedures) (Table 4). The most common vascular procedure was vascular suturing (22%, 34/156) followed by bypass/interposition graft (21%, 32/156). Vascular sutures were more frequent placed in the femoral vein (n = 5), the deep femoral artery (n = 4), and the inferior vena cava (n = 3). Repair with bypass/interposition graft was primarily performed in the lower extremity (26/32) followed by abdomen (3/32, iliacofemoral arterial bypass), upper extremity (2/33), and carotid artery (1/32). In the lower extremity, femoropopliteal bypass (n = 11) was dominating followed by bypass/interposition graft for the SFA (n = 9) and the popliteal artery (n = 3). Patch closure/vascular repair was performed more often for the brachial artery (n = 3), SFA (n = 3), and the common femoral artery (n = 2). Endovascular stent was placed in five patients (3.2%) (iliac artery n = 2, SFA n = 2, subclavian artery n = 1). Fasciotomy was performed in 17.9% (29/162) of patients. The frequency of reoperations was 8.6% (14/162); due to bleeding (n = 2), thrombosis (n = 2), bypass surgery (n = 1), and unspecified reason (n = 9). There were overall two amputations (1.2%; 2/162) including one transtibial amputation and one partial amputation of a finger.

**Table 4:** Vascular surgical procedures (n = 156) after firearm injuries in 74 patients

Vascular procedures	N (%)
Vascular suture	34 (21.8)
Bypass/interposition graft	32 (20.5)
Vascular exposure	32 (20.5)
Vascular repair/operation*	17 (10.9)
Thrombectomy	11 (7.1)
Angioplasty	7 (4.5)
Angiography	7 (4.5)
Vessel ligation	6 (3.8)
Endovascular stent	5 (3.2)
Miscellaneous vascular procedure	4 (2.6)
Coil embolization	1 (0.6)

*Patch closure/angioplasty or operative vascular repair not further defined

### Patient outcome

The 30-day and 90-day mortality was 29.9% (50/162) and 33.3% (54/162), respectively. The majority of deaths (79.6%; 43/54) was within 24-h of injury. For patients arriving in the ED with SBP < 90 mmHg or no palpable pulse; 43.1% (25/58) underwent thoracotomy and 31.0% (18/58) laparotomy; 24.1% (14/58) underwent both thoracotomy and laparotomy Seventy-four per cent (43/58) of patients with prehospital SBP < 90 mmHg/no palpable radial pulse died. In this hemodynamic unstable cohort, the need for laparotomy or thoracotomy was associated with a mortality of 77.8% (14/18) and 84% (21/25), respectively. Patients with prehospital cardiac arrest or intubation had an associated mortality of 97.3% (36/37) and 90.0% (27/30), respectively.

In the overall mortality group, injuries to the thoracic aorta (29.6%, 16/54) followed by the femoral (16.7%, 9/54) and carotid artery (13.0%, 7/54) were most common. The mortality in patients with injury to the thoracic aorta was 100% (16/16). The median number of days on mechanical ventilation was two ([IQR 1.0–5.0], n = 72). The median hospital length of stay was five days ([IQR 2.0–14.5], n = 159).

In the multivariate regression analysis, vascular injury to chest (*P* < 0.001) or abdomen (*P* = 0.002) and injury specifically to thoracic aorta (*P* < 0.001) or femoral artery (*P* = 0.022) were all associated with 24-h mortality.

## Discussion

Firearm injuries are a major global public health issue but there are few European studies. Research on a national level is imperative to increase knowledge of firearm injuries and to improve initial management. This population-based study showed an annual increase of firearm-related vascular injuries in recent years. Sixteen percent of all firearm injuries had at least one related vascular injury and predominantly affected young males. The mortality was high with almost a third of all patients dying within 30 days and approximately 80 percent of all deaths occurred within 24 h from the time of injury.

More than one third of patients arrived at the ED in hemorrhagic shock. In patients with hemodynamic instability, injuries to central vessels in chest and abdomen were more frequent. Several of these patients also required thoracotomy or laparotomy. These injuries are among the most difficult and challenging injuries to manage which was exemplified by firearm injury to the thoracic aorta where none survived. Damage control resuscitation and immediate open surgical hemorrhage control are the mainstay of treatment [10]. Emerging technology including resuscitative endovascular balloon occlusion of the aorta (REBOA) and endovascular interventions may be used as adjuncts to improve outcome [10]. Vascular damage control techniques including ligation of vessels should be used in complex vascular injuries. Ligation of major venous injuries in the abdomen or the lower extremity have not been associated in previous reports with mortality or lower extremity amputation [11–13].

The median transportation time was longer than half an hour for patients with hemodynamic instability. This may indicate a need for more advanced prehospital resuscitative care starting from the first patient contact [14]. The use of prehospital blood transfusion seems beneficial but an in-hospital survival benefit in hemorrhagic trauma patients is still unclear [15].

The mortality after firearm related vascular injuries was high and associated with non-compressible thoracoabdominal vascular injuries but also injury to the femoral artery. An overall mortality from abdominal vascular injuries of 54% has previously been reported [16]. The femoral artery injuries need further analysis and may be explained by junctional injuries where direct pressure or tourniquet may have been difficult to apply. This again emphasize the importance of rapid hemorrhage control and the use of vascular damage control techniques.

There were 7.3% of all patients with firearm injuries that required a vascular procedure. This is similar to a retrospective analysis from the US National Inpatient Sample where 9.9% of firearm injuries required a vascular repair [5]. The need for vascular repair in the US study predicted higher in-hospital mortality, sepsis, and any complication [5].

Gunshot wounds to the lower extremity remained the leading anatomic location and the femoral artery the most common vessel injury. Repair with bypass/interposition graft was primarily performed in the lower extremity. From the South African experience of penetrating femoral artery injuries, the superficial femoral artery (87%) was most commonly injured and about one third of patients were repaired with a vein interposition graft [17]. The in-hospital amputation rate in our study was low. Firearm-related penetrating injury to the lower extremity has in general a higher major amputation rate. Siracuse et al*.* reported an amputation rate of 3.3% compared to 0.8% for non-firearm penetrating trauma [6].

Endovascular stents were used in 3.2% of all vascular procedure and placed in the subclavian and iliac arteries, as well as in the SFA. Firearm-related vascular injuries are preferentially treated with open surgery and the endovascular therapy is primarily used among severely injured blunt trauma patients with noncompressible torso haemorrhage [18, 19]. The role of endovascular techniques in penetrating trauma needs to be further defined. The use of a trauma hybrid operating theatre may facilitate the combination of open and endovascular techniques in the management of firearm-related vascular injuries.

There are limitations inherent to this study including limited variables or missing data due to lack of registration or differences in registration practices. There may be a risk of missing patients due to lack of registration in the beginning of the implementation of the trauma registry more than a decade ago. However, the registry data were scrutinized for the three major trauma centers in the country and potentially missed registered patients with firearm-related vascular injuries were added. Fifty-three percent of coded vascular injuries required a registered vascular procedure. Some of the vascular injuries may have been managed with conservative treatment, direct pressure or hemostatic packing, and not with a specific vascular procedure, and underreporting of vascular suturing in operative notes may also have occurred. Moreover, pre-hospital use of tourniquet or administration of hemostatic agents were not available in the registry. Complications were limited to in-hospital stay but mortality data could be extended to three months. Furthermore, some variables might be subject to variations in practice due to coding. Standardized data techniques were used, and missing values imputed by combining numeric and ordinal values, to adjust for variation in data quality and completeness. The registry data was scrutinized for the three largest trauma centres in the country and potentially missed registered patients with firearm-related injuries and data on specific variables were added.

## Conclusion

This study explored firearm-related vascular injuries by assessing anatomical distribution, vascular procedures, and outcomes. The lower extremity and injury to femoral artery were the most common injury locations but vascular injuries to chest and abdomen being most lethal. A large portion of patients had hemodynamic instability. Early hemorrhage control combining open surgical and endovascular vascular techniques seems here critical for better outcome. There is a need for improved pre- and in-hospital management strategies but also for implementation of policy and public health efforts to reduce gun violence.

## Data Availability

Please contact author for data requests.
